# Phages Make for Jolly Good Stories

**DOI:** 10.3390/v10040209

**Published:** 2018-04-20

**Authors:** Thomas Häusler

**Affiliations:** Swiss Public Radio SRF, Redaktion Wissenschaft, Studio Basel, Novarastrasse 2, 4059 Basel, Switzerland; thomas.haeusler@srf.ch

**Keywords:** phage therapy, history of science, science communication

## Abstract

Phage therapy has an intriguing history. It was widely used from the 1920s until the 1940s. After this period, it was nearly completely forgotten in the Western world, while it continued to be used in the Soviet part of the globe. The study of the history of phage therapy provides valuable input into the present development of the field. Science journalists uncovered much of this history and played an important role in the communication of phage therapy after the fall of the Soviet Union, when it came to the attention of Western researchers and doctors. This interest was fueled by the antibiotic resistance crisis. At this time, communication about phage therapy had a wide potential audience, that encompassed medical experts and researchers, as well as the public, because knowledge about this forgotten therapy was very limited. In such a situation, good communication had and still has the potential to catalyze important discussions among different groups; whereas, bad communication could have considerably hindered and still can hinder the possible renaissance of phage therapy.

## 1. A Journalist’s Paradise

It is a core competence of journalists who are worth their salary to spot a good story. The phage therapy story that presented itself in the mid-1990s surely was one. A few years before, the Soviet Union had collapsed, and, slowly, information about a therapy that could tackle bacteria resistant to antibiotics began to trickle westwards. In view of the looming antibiotic resistance crisis this, in itself, would have been worthy of many “exotic therapy” reports for Western eyes at the time. However, there was a lot more to it. The therapy in question—called phage therapy—had been pioneered more than 70 years prior. It had been used extensively in many parts of the world until about World War II, when it was forgotten in the Western part of the world. Phage therapy lived on behind the “Iron Curtain” until the Eastern bloc disintegrated. Additional lure lay in the way the therapy worked, by using viruses to fight pathogenic bacteria.

There were even more ingredients for a perfect story with a lot of human interest. One of the important centers of Soviet phage therapy practice and research had been based in Tbilisi, the capital of the now independent state of Georgia. After independence, Georgia went through a great deal of turmoil, a short civil war, and huge economic problems. As a consequence, the phage researchers and their institutions in Tbilisi were in very dire straits and the staff had to subsist on a very meager pay. Most buildings were run-down, and the equipment in labs and hospitals was old, sparse and often broken. The walls lacked paint, floors were cracked, and many lights did not work. Peter Radetzky was, to the author’s best knowledge, the first Western journalist to travel to Tbilisi to report on all of this and must have felt in a journalist’s paradise. His 1996 article in Discover magazine [1] opened the way for more reporting.

## 2. To Hype or Not to(o) Hype?

Hypothetical observers of this state of affairs could have asked themselves: What will be the optimal type of reporting in the following years if we want to maximize the benefit of this discovery to society? Journalism has many functions, but this one question was chosen for the sake of this exercise. A first quick answer could have been: report it in a way that helps phage therapy achieve a quick global comeback. This answer would have been wrong. Research uncovered that the evidence for phage therapy’s effectiveness was not clear-cut at all.

Journalism textbooks provide ample messages of caution in such situations. Do not hype! Yet, there are quite a few examples from recent history where journalists, science communicators and scientists were guilty of exactly this. Think, for example, about the enthusiasm that greeted the first gene therapy attempts or the hypothesis that cancers could be cured by inhibiting angiogenesis. To be sure, both approaches eventually yielded important therapies and researchers continue to develop new ones. However, there were times when expectations were much higher than reality. These were fueled by over-optimistic assessments by scientists, and by journalists who uncritically featured them in their reports.

Several studies have shown that the main sources of exaggerations about scientific or medical findings are actually press releases issued by the institutions of the scientists that did the original studies [2,3]. Often, after inevitable roadblocks and setbacks have emerged, the reaction turns to the other extreme. The initial hype is followed by periods in which an approach is all but pronounced dead, as happened with both the examples mentioned above. It is a boom–bust cycle [4] that helps neither science nor journalism. How much hype and the boom–bust cycle actually damage the reputation of science as a whole or an individual discipline is debated in the literature. Many scholars argue that considerable damage can be done [5].

I would argue that phage therapy is especially vulnerable to such damage to its reputation due to its “exotic” history. However, there is a competition of ideas, research disciplines, and stories for the attention of the public, for funding and investments. To have a chance in this competition you need to make your case. Clearly, phage therapy has great potential, especially with regard to the antibiotics resistance crisis. Scientists and other actors in the field have to find the right balance between selling and overselling what they have to offer. The same holds true for reporters; they need to tell a good story and to give an accurate picture of the potential of their topic. How all actors have fared in this balancing act is discussed in different places in the text below. As a disclaimer, because I am an actor myself, I will mostly refrain from judgments and will instead try to highlight some of the aspects that I deem interesting.

## 3. Finding the Facts

Returning to phage therapy in the late 90s, it was not easy even for experienced reporters to find the fact base necessary to give an accurate picture. On the one hand, it seemed that if more than 70 years of research had not been able to prove the efficacy of the therapy beyond doubt, it was worth only very skeptical reporting. This seemed to be the order of the day. A skeptical journalist is, of course, a good journalist. However, perhaps depicting phage therapy as a fascinating yet utterly obsolete method was not the best way to go about it. Maybe the therapy did have the potential to help combat antibiotic resistance and reports that were too negative mounted additional barriers for a struggling, yet helpful, medical therapy?

For reporters, this difficult situation lasted well into the 2000s. At the time, little information was forthcoming. Most of the scientific literature was buried in pre-medline repositories, such as a long-forgotten, hand-collected literature list [6,7] that a German phage researcher collected and published in the 1950s and 1960s (11,405 references, painstakingly listed in two two-tome publications, and painstakingly read by the author of this article to be able to track down as many interesting articles as possible). Much of the phage literature concerning medical therapy was written in Russian or Georgian, which made it even more difficult to obtain and read. Ideally, a reporter would travel to Georgia and see this research for themselves. However, not many could do this, given that they had to find an editor willing to produce the funds for such an investigation. Once in Georgia, the reporter could meet with phage researchers and doctors who warmly testify in favor of the method. Georgia’s many troubles after independence cast most research institutes and hospitals in a sorry state. It was obvious that researchers and doctors did an admirable job to keep things running. Nevertheless, this situation did not instill a lot of conviction in a visitor who came looking for evidence for a therapy that was co-developed here.

The reporter’s resolve to keep an open mind was further weakened by researchers from Western institutions uttering pungently negative views [8]. Often, these seemed influenced by the renowned phage researcher Gunter Stent. In his 1963 textbook “Molecular Biology of Bacterial Viruses” he offered the assessment that phage therapy was not working, because the human immune system removes phages, stomach acid destroys them, and bacteria become resistant to the phages applied in therapy. Stent was quite adamant in his negative opinion, as this quote from the book attests: “The strange bacteriophage therapy chapter of the history of medicine may now be fairly considered as closed” [9].

Another line of reasoning feeding into the negative view was the very skeptical opinion of science conducted in the Soviet Union [8,10]. This view might have been based on strong indications; however, it was not bona-fide proof that phage therapy did not work. Yet, all these factors cast their shadows into the 1990s and early 2000s, when Western scientists became aware of the vast practice of phage therapy in the Soviet Union. An interesting impression of how many Western scientists thought about the field at the time is given by a quote by Ry Young (Center for Phage Technology, Texas A&M). He stated, in 2017, in a phage workshop hosted by the US Food and Drug Administration (FDA): “In fact, since I was up until quite recently biased by my training in the phage group [a group of scientists that, starting in the 1940s, studied the molecular biology of phage], I was actually very anti-phage therapy” [11].

## 4. In the Age of Story Telling

Humans have probably told each other stories since they started to talk. However, it seems that we are living in an era in which story telling as a concept receives renewed and intense interest, in journalism [12], in science communication [12,13], and in science [14,15]. Journalism basically thrives on the millennia old wisdom that nothing captivates people more than a good story. However, even in science, journal editors and readers alike favor a good story; a set of experiments that fit nicely together into one narrative and that convey a coherent picture of a sub-set of reality. There is nothing amiss with this. The chemist and Nobel Laureate, Roald Hoffmann, even argues that using story-telling in a scientific paper can help to convey complex research findings or concepts to fellow scientists [15], and an analysis of scientific publications dealing with climate change has shown that publications with more narrative abstracts are cited more often [16]. However, if everybody is tuned to “good stories” and to coherent narratives, readers might have less patience for stories that are more complex or look to much into the underlying complex reality. They might even mistrust stories that do not fit a clear-cut narrative.

Does phage therapy work or does it not? Maybe it works, but only under certain circumstances. Scientists should be prepared for such a reality. There are plenty of established examples of drugs that work for patients with a certain genetic background, for example, but not for others. In the case of phage therapy, for instance, bacteria infecting a patient can develop resistance against a phage used for treatment [17]. In this case, an alternative phage or phage cocktail has to be used to continue treatment. Of course, scientists and doctors working in the field are well aware of this and other potential obstacles. However, every scientist is not an expert in all disciplines except their own. This may sound like a truism, but it is also a hint that all of us will not necessarily approach foreign disciplines with the usual scientific rigor and an open mind.

In the early days of renewed Western interest in phage therapy, at least one thing seemed clear: there was a huge amount of ignorance about the subject across most groups one could think of. This included the general public, most scientists and doctors, the pharmaceutical industry, the intellectual property community, investors, drug approval agencies, and so on. If phage therapy stands a chance at making a comeback, the education of all of these stakeholders is important. Scientists and doctors are necessary to create evidence that the therapy works. The pharmaceutical industry and investors are needed to fund the research and bring phage drugs to market. Since phages are natural entities that have been long-known to science, the patenting of drugs is not straightforward at all. Drug agencies need to explore the ways in which a preparation of viable viruses could be fed into the drug approval process. More will be discussed on this point later.

The researchers and practitioners of phage therapy in Tbilisi and other parts of the former Eastern bloc (namely Poland and Russia) knew, of course, a lot about the core topic but they needed knowledge about other matters, namely drug development, business development, intellectual property issues and many more.

How large the dangers of a bipartite information deficit was, became quickly and painfully clear. Radetzky’s 1996 article catalyzed the first contact between Western investors and phage researchers in Tbilisi. A Canadian financier visited the city and quickly struck a deal with some of the researchers of the Eliava institute. However, things ended in bitter conflict. The US start-up set up to develop a phage drug for the Western market soon pulled out of the co-operation with Tbilisi. The reason for this, reportedly, was that the difficult circumstances in Georgia might taint the reputation of the future drug. The phage researchers in Tbilisi felt cheated. Additionally, the contract with the start-up created much tension among different groups of the Eliava Institute [18]. In 2003, a manager of the US start-up was quoted in the media with very negative statements about the work of the phage researchers in Tbilisi: “They made every mistake in the book and did some really stupid things. […] They just took what was on the shelf and assumed it would work.” [19]. At this still very early stage of rediscovery of phage therapy in Western countries, such a statement ran a very high risk of tainting the reputation of the whole field severely.

## 5. History’s Worth

It turns out that much can be learned from extensive research into past literature. There are studies covering hundreds of thousands of subjects treated with phages, as well as many different types of infections and species of bacteria. Due to the fact that most of these studies were done before the 1960s, they do not hold up to the standards of the modern era, of double-blind, randomized, controlled clinical trials. There is a lot of information that can be found, nevertheless. For example, there is enough information to instill interest in potential investors and to help gauge the medical potential of the therapy.

Analysis of the past literature also provides reasons for why phage therapy was nearly completely forgotten after World War II in Western countries. One reason surely had to do with the fact that, even for the time, many practitioners and researchers were neglecting scientific rigor and standards significantly. This led to much-deserved criticism and skepticism, for instance in the form of several derogatory editorials in the Journal of the American Medical Association [10,20,21,22]. Another reason was that knowledge about the basic biology of phages was still rather limited.

Ironically, this kind of trouble had been foreshadowed with uncanny precision by the US writer and Nobel Laureate, Sinclair Lewis, in his 1925 bestseller, Arrowsmith [23]. In his novel, Lewis heavily criticized the way in which medical researchers and doctors were business-minded. At the center of his story, is a phage therapy trial in the Caribbean. His hero, Martin Arrowsmith, had travelled to an island where plague raged. He had all intents to do a properly controlled study by treating only half of the sick with a phage against *Yersinia pestis*. The other half was to receive a placebo. Then, Arrowsmith’s wife died from the plague, and in his grief, he let slip all his resolve and discipline for protocol and treated all patients alike with the phage.

## 6. Communication in Times of Slow News

Lewis’ pen was guided by the scientist-turned-science-writer, Paul de Kruif. Together, they crafted a story with a shadow that reached into the era of phage therapy’s potential renaissance. After the “Iron Curtain” came down, the full comeback of phage therapy was, and still is, slow to materialize. What is needed are successful clinical trials. Some Phase I and II trials have been conducted but, to date, no Phase III trial has ever been initiated. Arrowsmith’s great aim has still not yet been achieved. Medical progress is often slow in the making, and obstacles are part of the business. However, phage therapy seems to fight with more than its fair share of obstacles [24]. The reasons are manifold, but the most important is probably that the way phage therapy could be made to work does not fit within the current paradigms of the pharma market.

This poses special difficulties for the actors in the field of phage therapy, as far as communication is concerned. Slow progress means that little news is forthcoming that could be interesting to the public. In this situation, some actors could be tempted to hype their work to garner attention. Indeed, this has happened in some instances. In one case, the media department of a university issued a release about a study using phages against bacterial infections in Cystic Fibrosis (CF) lung infections [25]. The title read “Phage therapy shown to kill drug-resistant superbug”. The release discussed the severe problem of lung infections in CF patients and stated: “Here for the first time, researchers have shown that phage therapy is highly effective in treating established and recalcitrant chronic respiratory tract infections caused by multi-drug resistant *Pseudomonas aeruginosa* strains. They show that phages are capable of killing the bacteria in long term infected lungs, such as those suffered by patients with the inherited disease Cystic Fibrosis, indicating a potential new therapeutic option for these hard to treat life threatening infections”.

Careful readers will wonder if actual patients have indeed been treated, but the casual reader will probably presume so. The media release did not help readers perceive the research clearly, as it never mentioned that the experiments were done exclusively in mice and in an artificial model of a lung [26]. The plight of CF patients is well known. Many of them would jump at such a news and presume that help for their infection is just around the corner. Judging by the information contained in the original paper, this is not the case. However, the media department release was taken up virtually unchanged by a widely read internet site publishing popular science articles [27].

A quantitative survey of reports regarding phage therapy is outside of the scope of this article. I have surveyed, approximately, the first 50 articles that show up in a Google News search with the term “phage therapy”. Based on this, it is fair to say that this kind of communication, by scientists or university media staff, is the minority. Many researchers seem to do an excellent job in providing a balanced picture. For example, in 2016, a diverse group of US researchers and doctors treated a patient that was suffering from a very severe *Acinetobacter baumannii* infection with phages. As is the nature of anecdotal cases, there was no definitive proof that the phages really cured the patient but the probability seemed quite high [17]. This publication triggered a host of reports in the press. Several aspects made the case intriguing. The patient was saved from what seemed to be sure death and he was treated with phages intravenously. Nevertheless, articles about the case were generally of a high quality.

Obviously, the scientists and media staff of their institutions did a good job. They provided extensive material for the media [28], and the scientists positioned the nature of a single case study very clearly when they were interviewed, stating, for example, “But for now, his case is just an anecdote—albeit a hopeful one—as all the physicians and scientists who worked on his case point out.” [29]. This constructive communication was remarkable, since quite often the media overblow the significance of such anecdotal stories of healing, giving the impression that a single cured person constitutes proof of effectiveness.

This example shows the chance that lies in such a story with a human dimension if the science involved is communicated well. It triggered extensive news coverage, which is quite a success for a medical therapy that, in the eyes of a hard-nosed news editor, has not been making significant news for quite some years. The renewed media coverage may, in turn, have generated renewed interest from the grant and investment community. Intriguingly, the reports were quite long and detailed, not only in the aspects of human interest but also in the science. For instance, they mentioned the fact that the bacteria causing the infection developed resistance and that to continue treatment new phages had to be found. In this way, the reports conveyed a picture of the complexity of phage therapy, which helps to explain to the public why it is taking so long to develop this method into an established medical treatment.

Phage researchers could potentially learn still more from colleagues in other disciplines that have a lot of experience with communication. Climate science springs to mind because it is also a field that needs to communicate a lot with the public, and over a long period of time. Some climate scientists have devised a strategy of building a certain basic consensus among scholars to provide clearer communication to the public [30]. This has been taken up by the media quite extensively. Perhaps this concept could be adapted to the field of phage therapy.

## 7. Giving a Balanced View

Section 6 looked mainly at the role of scientists in the way facts about phage therapy are communicated. What about journalists? Ideally, journalists should be able to give a balanced view of the potential of phage therapy and the obstacles it faces. In the majority of cases that came out of the Google News search mentioned above, journalists did a good job. They usually mentioned that anecdotal cases are not general proof of efficacy, for example. However, some fail to provide this important aspect. Others offered general statements that were incorrect without proper specifications: “Even though bacteriophages […] work better than antibiotics […]” [31]. Many ran titles that seemed to talk of a miracle cure (“Bacteriophage therapy, the amazing cure for MRSA being ignored by mainstream medicine“), and this initial impression was never put into perspective or it was, but only very late into the article, when many readers probably had long escaped the text [28].

Whether the picture regarding communication quality overall is rather bright grey (as I think) or a bit darker, is up for personal judgment. Of course, there is always room for improvement. Platforms like healthnewsreview.org are trying to tap into that potential. Journalists provide critical appraisals of selected media articles and point out weaknesses. Up until now, they have done so with one article concerned with phage therapy [32].

## 8. Patience Is Still Required

What about the way forward? Again, a glance back in time might help. It turns out that the curative powers of phages were not completely forgotten in the West. Dedicated scientists, doctors and small companies kept it alive until the 1960s and 1970s, for example in France and Switzerland [33,34]. The way it was practiced there hinted at possible schemes that could be used today. Researchers were keeping a large and diverse bank of phages that could be used to search for effective variants when a patient presented with an infection. The concoction used was produced on demand. There was no or only a small profit involved.

This kind of scheme was again proposed by several actors in the early 2000s. In Georgia, it has been used for the last 80 years, and has a long tradition in Poland, as well. There is still some work required before it can be applied in other European countries or the US. However, after long attempts to bring phage therapy to market using more conventional approaches, there seems to be a willingness by several stakeholders to explore avenues that are unconventional relative to the regulatory framework in Western countries. In 2015, for instance, researchers, physicians, and industrial representatives of several countries met in Tbilisi to discuss how phage therapy could be used in Western health settings [35]. In the same year, the European drug regulatory agency EMA convened a workshop with representatives from industry, academia, regulatory authorities and legislators to discuss similar questions [36]. In 2017, the FDA held, as well, a workshop to discuss scientific and regulatory issues [37].

At this workshop, a representative of a French company that produces phages according to an officially regulated standard (“Good Manufacturing Practice”-production (GMP)) for clinical research, mentioned that these phages obtained clearance by the French authorities to be used outside the clinical trial in cases where patients are critically ill [38]. Belgian health authorities are preparing an approach that paves the way for the small-scale use of phage therapy [39]. The basis for this is a central phage bank, with controlled deposits that serve as a source for treatments.

There is still work to be done. Solid proof for phage therapy’s effectiveness remains patchy [40]. Laudable initiatives like the EU-funded PhagoBurn study have been initiated, but have apparently run into difficulties [41]. If the fascinating history of phage therapy is any guide, this is not surprising. Yet, there seems to be, once again, renewed activity in the field. Amongst other developments, the US Navy is working on phage therapy [17], the German government is funding a phage project (“Phage4Cure”), and in the US, scientists have set up a virtual phage bank to help quickly source therapeutic phages for urgent needs [42]. If journalists, with their research and analysis [43,44], have had even a small catalytic function in this development, it would have been well worth the considerable investment.
